# ST-Segment Elevation Myocardial Infarction Caused by 5-Fluorouracil-Related Cardiotoxicity

**DOI:** 10.7759/cureus.52864

**Published:** 2024-01-24

**Authors:** Nivedha Balaji, Priyadarshini Dixit, Alex M Adams, Fardeen Faiz, Daisy Ngwainmbi, Glen Henry, Nima Ghasemzadeh

**Affiliations:** 1 Internal Medicine, Northeast Georgia Medical Center Gainesville, Gainesville, USA; 2 Cardiology, Northeast Georgia Medical Center Gainesville, Gainesville, USA; 3 Graduate Medical Education, Northeast Georgia Medical Center Gainesville, Gainesville, USA; 4 Interventional Cardiology, Georgia Heart Institute, Gainesville, USA

**Keywords:** chemotherapy-related toxicity, interventional cardiologist, 5fu, st-elevation myocardial infarction (stemi), #cardio-oncology

## Abstract

5-Fluorouracil (5-FU) and its prodrug, capecitabine, are commonly used chemotherapeutic agents for solid tumor management. While these agents can present with adverse side effects such as nausea, vomiting, diarrhea, and myelosuppression, they can also, less commonly, cause cardiovascular toxicity. This toxicity may manifest as cardiac arrhythmias, myocarditis, heart failure, myocardial infarction, and even death. The management of 5-FU-related cardiotoxicity includes early recognition of symptom manifestation so that medication can be discontinued promptly and symptoms can be addressed appropriately. Here, we describe the case of a 72-year-old male who developed coronary vasospasm and ST-segment elevation myocardial infarction shortly after the initiation of chemotherapy with 5-FU.

## Introduction

5-Fluorouracil (5-FU) is the third most used chemotherapy worldwide and is the second most associated drug with cardiotoxicity [1]. 5-FU and its oral prodrug, capecitabine, are commonly used in solid tumor management, including head and neck, bladder, breast, and gastrointestinal malignancies [1,2]. As a thymidylate synthase inhibitor, 5-FU impedes the synthesis of thymidine and DNA replication [2]. This chemotherapy agent may cause a range of side effects, such as nausea, emesis, diarrhea, mucositis, alopecia, myelosuppression, hand-foot syndrome, and cardiovascular toxicity [2]. Manifestations of 5-FU-induced cardiotoxicity include chest pain with or without exertion, atrial fibrillation, myocarditis, pericarditis, heart failure, acute coronary syndromes including myocardial infarction, and even death [1]. We present a case of a 72-year-old male with a history of colon cancer on 5-FU who presented with ST-segment elevation myocardial infarction (STEMI).

## Case presentation

**Figure 1:**
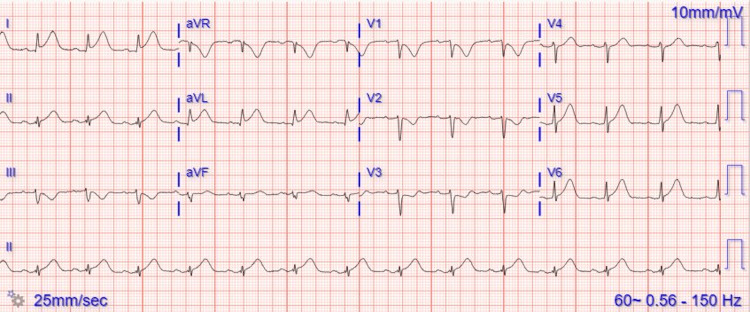
Electrocardiogram showing high lateral ST elevations in lead I and aVL.

**Figure 2:**
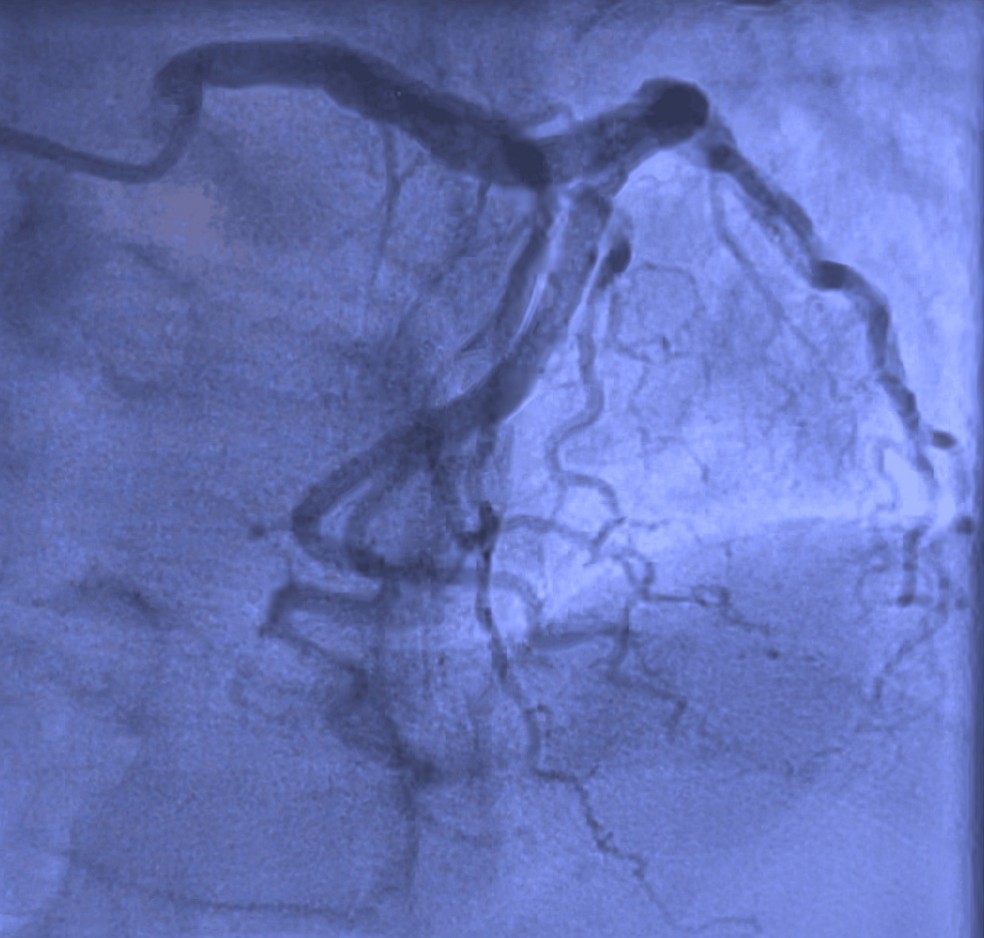
Coronary angiography of the left system demonstrating non-obstructive epicardial coronary disease.

**Figure 3:**
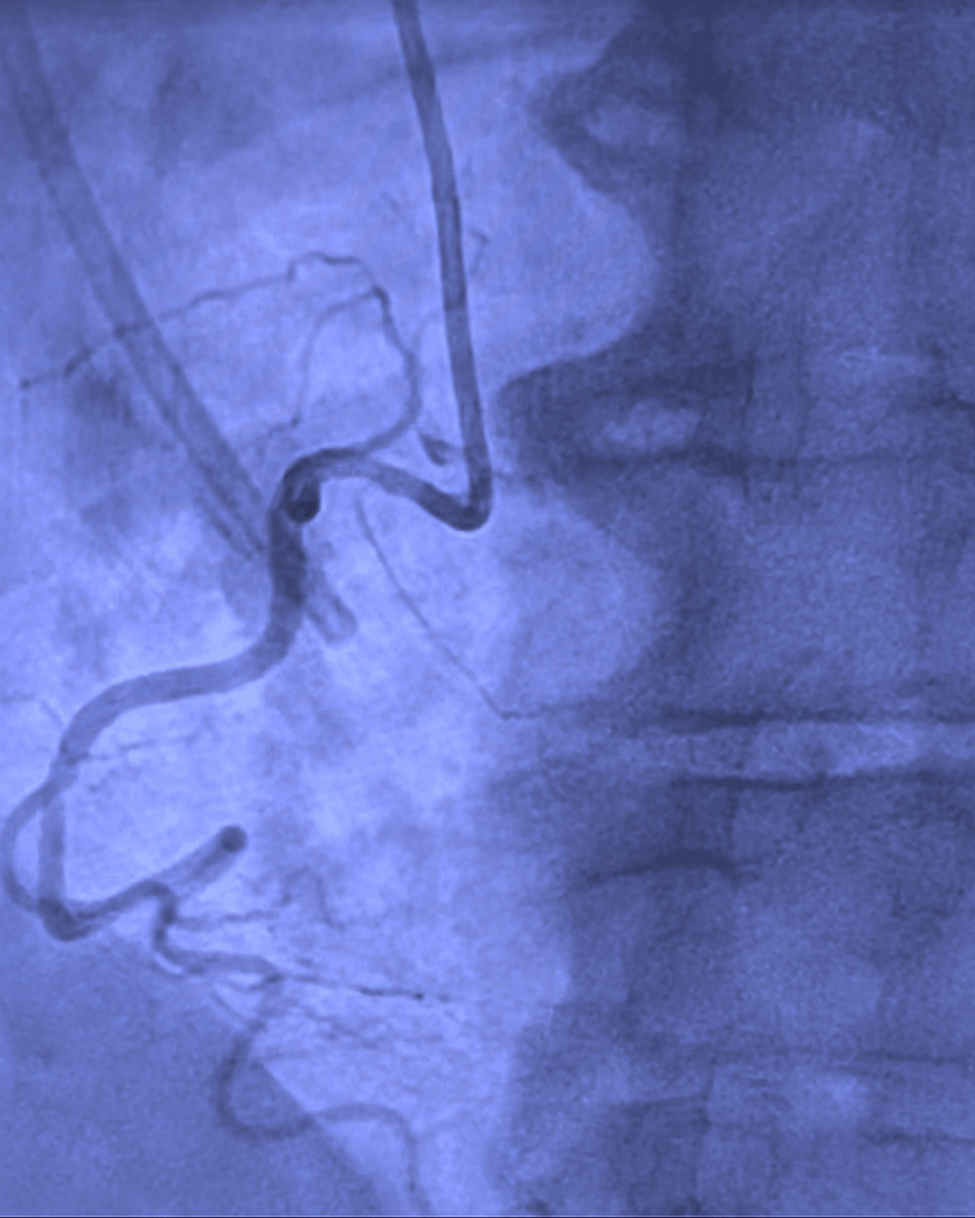
Coronary angiography of the right system demonstrating non-dominant right coronary artery.

A 72-year-old Caucasian male with a medical history of hypertension and colon cancer on 5-FU presented to the emergency department (ED) with chest pain that began a few hours earlier that morning. The pain was intermittent, moderate in nature, non-radiating, substernal, and associated with diaphoresis. Notably, he had recently started his first round of chemotherapy, with the chemotherapy port placed just two days before his hospital presentation. Vital signs revealed a heart rate (HR) of 60 bpm, blood pressure (BP) of 140/70 mmHg, and an oxygen saturation of 95% on room air. Cardiac examination revealed normal S1/S2 and no murmurs. Laboratory values on presentation are depicted in Table 1. Initial high-sensitivity troponin was 20 ng/L but peaked at 110 ng/L and subsequently trended down. An electrocardiogram (EKG) showed high lateral ST elevations in lead I and aVL (Figure 1). He was then taken to the cardiac catheterization laboratory for emergent coronary angiography. Via the right radial approach, coronary angiography demonstrated non-obstructive epicardial coronary artery disease with no angiographic evidence of significant stenosis, plaque rupture, or thrombus (Figures 2, 3 and the Appendix). A transthoracic echocardiogram showed an LVEF of 50-55% with no regional wall motion abnormality and no significant valvular abnormality. The angiographic evidence raised suspicion for another underlying etiology for his clinical presentation.

**Table 1:** Table demonstrating significant laboratory values upon initial presentation BUN: blood urea nitrogen.

Laboratory tests	Value	Reference range
Hemoglobin	11 g/dL	13-18 g/dL
White blood count	11.4 x 10^3^/mL	4.0-11 x 10^3^/mL
Platelet count	191 x 10^3^/mL	150-400 x 10^3^/mL
BUN	29 mg/dL	7.0-20 mg/dL
Creatinine	1.54 mg/dL	0.6-1.2 mg/dL
High-sensitivity troponin	110 ng/L	<14 ng/L
Low-density lipoprotein	100 mg/dL	<100 mg/dL

A further detailed history was obtained, and the patient stated that he received his first session of chemotherapy with 5-FU for his colon cancer one day before presentation. The underlying etiology for the STEMI was thought to be coronary vasospasm caused by the recent initiation of this chemotherapy agent. A discussion was held with the patient's oncologist, and a shared decision was made to avoid 5-FU in the future given the current clinical presentation.

The patient was started on verapamil SR 120 mg once a day for coronary vasospasm. 5-FU was then discontinued, and he was discharged home in a medically stable condition after being monitored for 48 hours. Apart from the calcium channel blocker, additional medications upon discharge included isosorbide mononitrate 30 mg once a day and atorvastatin 40 mg once a day. At his follow-up appointment, he did not endorse any more episodes of chest pain and tolerated verapamil SR 120 mg daily well, which was continued.

## Discussion

Approximately up to 5% of patients treated with fluoropyrimidines like 5-FU or capecitabine may experience symptoms of cardiovascular toxicity [3]. No reliable data has shown existing cardiac comorbidity to be a predisposing factor in the development of cardiotoxicity in patients treated with fluoropyrimidines. It is estimated that over 50% of patients who develop cardiotoxicity have no prior history of cardiac comorbidity [4]. Previous studies have demonstrated that while pre-existing heart conditions may serve as a risk factor for cardiotoxicity, they are certainly not a prerequisite [5]. Risk factors of fluoropyrimidine-induced cardiotoxicity include the concomitant use of polychemotherapy and the administration of radiotherapy. Studies have shown that continuous dosing of 5-FU is associated with an increased incidence of cardiovascular toxicity compared to bolus dosing [2].

The mechanism behind 5-FU cardiotoxicity, although unknown, is likely multifactorial, involving coronary vasospasms-induced myocardial ischemia and drug-related myocardial injury [1]. Vasospasm can present in the setting of endothelial or smooth muscle dysfunction, characterized by an abnormal vasodilatory response or vasoconstriction. Pharmacologic provocation testing using nitroglycerin can assess coronary artery structure and endothelial function. Patients experiencing coronary vasospasm may exhibit ST-segment elevation on electrocardiogram and troponin elevation in the absence of occlusive vascular disease on imaging. Another theory for cardiotoxicity is direct cellular damage caused by dose-dependent injury to endothelial cells. This is thought to be due to hypoxic cell injury from 5-FU-induced mitochondrial uncoupling and reduced aerobic efficiency [1, 6]. However, there are inconsistencies with the theory of 5-FU-induced vasospasm causing cardiotoxicity. Vasoconstriction is noted in patients during or immediately after the administration of 5-FU, but symptoms of cardiotoxicity typically do not manifest until after the infusion or several hours to days later. Additionally, coronary vasospasm is not consistently observed via angiography in patients exhibiting symptoms of cardiotoxicity post 5-FU [6].

Approximately 44-90% of patients who initially develop cardiotoxicity can have a recurrence of symptoms with dose reduction or re-challenge of medication, even while receiving prophylactic cardioprotective medications [4]. The temporal association of symptoms and the administration of 5-FU must be assessed and correlated [4]. 5-FU-related cardiovascular toxicity can occur during medication administration, and capecitabine-induced cardiotoxicity can occur after three days of medication use [1, 3]. When toxicity is evident, it is essential to discontinue the offending agent and provide prompt symptomatic management with anti-anginal agents like calcium channel blockers and nitrates. Recognizing and changing treatment courses has been reported to alleviate cardiotoxicity symptoms in almost 69% of patients [6].

In one case series, 11 patients were re-challenged with fluoropyrimidine with simultaneous antianginal medications under cardiac monitoring and successfully completed their infusion [6]. A retrospective European study conducted between 2011 and 2020 concluded that S-1, an alternative fluoropyrimidine to 5-FU, is a safe and feasible therapy to be used after the development of cardiotoxicity with 5-FU. They demonstrated that S-1 can be used to treat solid tumors where fluoropyrimidine-based management is recommended, and that S-1 had reduced the recurrence rate of cardiotoxicity [4]. Clinicians may consider using alternate chemotherapy regimens for treating these patients. Another strategy may be dose reduction or prophylactic treatment with anti-anginal medications [6].

## Conclusions

In conclusion, recognizing the prevalence and symptom manifestations of cardiotoxicity from 5-FU is essential. The frequent use of fluoropyrimidines in treating many common malignancies, such as gastrointestinal and breast cancer, reiterates the importance of understanding the side effect profile of 5-FU. While the mechanism of fluoropyrimidine-related cardiovascular toxicity is poorly understood, symptoms may arise from coronary vasospasm and direct myocardial injury. Although there is no antidote for patients with toxicity from fluoropyrimidines, patients can be treated with medications to manage symptoms and reduce morbidity and mortality. Further studies are needed to understand the mechanism and management of 5-FU-related cardiotoxicity better.
